# Clinical trials of dual-target CAR T cells, donor-derived CAR T cells, and universal CAR T cells for acute lymphoid leukemia

**DOI:** 10.1186/s13045-019-0705-x

**Published:** 2019-02-14

**Authors:** Juanjuan Zhao, Yongping Song, Delong Liu

**Affiliations:** The Affiliated Cancer Hospital of Zhengzhou University and Henan Cancer Hospital, 127 Dongming Road, Zhengzhou, 450008 China

## Abstract

The current treatment for pediatric acute lymphoblastic leukemia (ALL) is highly successful with high cure rate. However, the treatment of adult ALL remains a challenge, particularly for refractory and/or relapsed (R/R) ALL. The advent of new targeted agents, blinatumomab, inotuzumab ozogamycin, and chimeric antigen receptor (CAR) T cells, are changing the treatment paradigm for ALL. Tisagenlecleucel (kymriah, Novartis) is an autologous CD19-targeted CAR T cell product approved for treatment of R/R B cell ALL and lymphoma. In an attempt to reduce the relapse rate and treat those relapsed patients with antigen loss, donor-derived CAR T cells and CD19/CD22 dual-target CAR T cells are in clinical trials. Gene-edited “off-the-shelf” universal CAR T cells are also undergoing active clinical development. This review summarized new clinical trials and latest updates at the 2018 ASH Annual Meeting on CAR T therapy for ALL with a focus on dual-target CAR T and universal CAR T cell trials.

## Background

The current treatment for pediatric acute lymphoblastic leukemia (ALL) is highly successful with cure rate approaching 80% [1–3]. However, the treatment of adult ALL remains a challenge, particularly for refractory and/or relapsed (R/R) ALL [4–9]. The prognosis of adults with R/R ALL is still very poor. The CR rate for R/R ALL has remained only 29% (range 18 to 44%), and the median overall survival (OS) is only 4 months (range 2–6 months). Novel agents to improve the outcome of R/R ALL are urgently needed. In recent years, tyrosine kinase inhibitors (TKI) have contributed to improvement of outcome of ALL with Philadelphia chromosomes (Ph+ALL) [10–17]. In the past few years, immunotherapeutic agents including blinatumomab and inotuzumab ozogamicin have been shown to increase response rate and extend OS in patients with R/R ALL [18–38]. Another significant advance in ALL therapy came when chimeric antigen receptor (CAR)-engineered T cells were approved by FDA for children and young adults with R/R ALL [39–46]. However, loss of antigen target has been reported to be a common mechanism for relapse after CAR T cell therapy [47–51]. In an attempt to reduce the relapse rate and treat those relapsed patients with antigen loss, donor-derived CAR T cells and dual-target CAR T cells are in clinical trials. Gene-edited “off-the-shelf” universal CAR T cells are also undergoing active clinical development [52–59]. More versatile and programmable CARs are being developed [59–62]. This review summarized new clinical trials and latest updates at the 2018 ASH Annual Meeting on CAR T therapy for ALL with a focus on dual-target CAR T and universal CAR T cell trials.

## CD19-targeted CAR T cells

### Long-term outcome of CAR19 T cell therapy for R/R ALL

CARs are engineered to bind to a specific antigen leading to activation of the CAR T cells without the dual restriction traditionally conferred by specific T cell receptor and the major histocompatibility complex (MHC) [42, 43, 63–69]. CD19 is the most common target of CAR T cells to date [46, 70–73]. Tisagenlecleucel (tis-cel) (kymriah, Novartis) is an autologous CD19-targeted CAR T cell product approved for the treatment of R/R B cell ALL and non-Hodgkin lymphoma (NHL) [48, 49, 74–76]. Another CAR T cell product targeting CD19 antigen, axicabtagene ciloleucel (yescarta, Kite), was approved for treatment of R/R diffuse large cell lymphoma [50, 77–79]. To date, two distinct CAR T-associated toxicities (CARTox) are cytokine release syndrome (CRS) and CAR T-related encephalopathy syndrome (CRES) [80–83]. Prophylaxis and therapy for CARTox are important areas of pre-clinical and clinical research [80, 81, 84].

Recently a multicenter phase II study of tis-cel CAR T cell therapy for children and young adults with R/R B-cell ALL was updated [49]. This update from the multicenter international trial reported a CR rate of 81% and the severe CRS rate of 77%. The 1-year EFS was 50%. With a median follow-up of 13.1 months, the median survival of these patients had not been reached. Tis-cel contains a CAR with 4-1BB as the costimulatory signal. The 4-1BB costimulation domain is known to be associated with longer persistence of CAR T cells and less T cell exhaustion. The tis-cel T cells were found to have an ongoing persistence of 20 months at the time of the report. It is known that higher leukemia burden is associated with higher CARTox, and CRS is associated with response, yet no linear relationship between CAR T cell dosage and response was observed.

The data from long-term follow-up of a single-center phase I study using 19-28z CAR T cell therapy for adult R/R ALL were updated in early 2018 [85]. The primary endpoint of this phase I study was safety. This study enrolled 75 patients (53 evaluable). The 53 evaluable patients have failed multiple prior therapies including prior allogeneic stem cell transplantation (allo-HSCT), blinatumomab, and TKIs in Ph+ALL. This study had a median follow-up of 29 months (range 1–65 months). The CR rate was 83%, and the median OS was 12.9 months. Low disease burden correlated with better outcome. Among patients with a low bone marrow blasts (< 5%), the median OS was 20.1 months. The rate of severe CRS was 26%. The CARTox after 19-28z CAR T-cell infusion was lower in patients with low disease burden as compared with those bearing a higher disease burden.

The Seattle group recently reported long-term adverse events after CD19-targeted CAR T cell infusions [86]. These events occurred or persisted beyond 90 days after the last CAR T cell infusion. Events related to disease progression were excluded. A total of 59 patients with R/R NHL and chronic lymphocytic leukemia (CLL) were included in this update. These patients survived more than a year and had at least 1-year complete follow-up data after their first CAR T cell infusion. At this report, the median follow-up was 23 months (range 13–57). These were adult patients with median age of 60 years (range 34–73). These patients had a median number of 4 lines of treatment (range 1–8). Among the 59 patients, 39% failed prior autologous (auto) hematopoietic cell transplantation (HCT), and 15% failed prior allogeneic (allo) HCT. Interestingly, severe CARTox was not seen in these patients with CLL and NHL, with CRS grade I/II, 64%; grade III, 7%; grade IV, none; and acute neurotoxicity, 34%. Cytopenias requiring G-CSF and/ or transfusion support were among the notable long-term adverse events. In addition, 41% of the patients were reported to have severe hypogammaglobulinemia (IgG < 400 mg/dL, or immunoglobulin replacement). Eight patients developed secondary malignancies, including 3 myelodysplasia, 4 non-melanoma skin cancer, and 1 non-invasive bladder cancer. It was noted that all except one with skin cancer had auto or allo HCT before CAR T cell therapy. Neuropsychiatric disorders, cerebro- and cardiovascular events, renal dysfunction, and respiratory disorders were among the reported long-term effects. Infection was reported in 74% of the patients, with the most common ones being the upper and lower respiratory tract infections. These long-term follow-up data confirmed that CD19-targeted CAR T cell therapy for NHL and CLL is well tolerated. Most long-term effects in these cohorts may have been related to prior or subsequent therapies since these patients have been heavily pre-treated.

These long-term outcome analyses of CAR T 19 cell therapy for R/R ALL clearly documented the efficacy of the CAR T cells in R/R ALL. Although there still lacks parallel comparison of CAR T cells and antibody-targeted therapies for R/R ALL, CAR T cells appear to have more durable response [71]. The 4-1BB containing CAR T cells appear to have relatively more durable response as well as CAR T cell persistence.

### CAR19 T cells used as reinduction/bridging therapy prior to allo-HSCT

In a recent report at the 2018 ASH Annual Meeting, CD19-targeted CAR T cells were administered to 83 patients with R/R ALL with a median age of 10 years (range 2–61) [87]. High-risk patients were enrolled in this cohort, including 17 with extramedullary disease (EMD) (11 with CNS disease), 10 with BCR-ABL+, 8 with TP53 mutation, and 11 with relapsed ALL after allo-HSCT. The second-generation CARs contained either a CD28 (*n* = 21) or a 4-1BB (*n* = 62) co-stimulatory domain. The patients received a single infusion of CAR T cells with a median dose of 1 × 10^5^ (0.1–10 × 10^5^) cells/kg. The median follow-up was 172 (27–325) days. The day 30 CR/CRi rate was 76/83 (91.6%) with 70/76 (92.1%) of them negative for MRD. One-year OS was 76.5% and RFS was 62.6%. CARTox included 16% grade III–IV CRS and 12% severe CRES. There was one CAR T-related death. CR was observed in 14/17 of patients with EMD, including CR in 9/11 patients with CNS leukemia. OS was significantly longer in those without EMD (45.1% vs 88.3%, *p* = 0.0003). Consistent with the literature, OS was significantly better in patients with low leukemia burden (LB) (46.2% high LB vs. 7.1% low LB, *p* = 0.001). Even though no difference in CR/OS/RFS between patients with or without the BCR-ABL mutation was observed, the small sample size makes it impossible to claim that the low-dose CAR T therapy negated the poor prognostic factor of BCR-ABL mutation. Even though the CR/CRi was achieved in 7/8 patients with TP53 mutation but relapse rate was high. The OS and RFS were significantly lower for patients with TP53 mutation. The CR/CRi rate after CAR T infusion was 91% in the 11 patients who relapsed after allo-HSCT. No significant GVHD was observed. Among the 76 patients with day 30 CR/CRi, 57 proceeded to allo-HSCT in a median time of 2 months post CR. The 1-year OS of the CAR T-to-allo-HSCT group was better than that of the non-transplant group (OS 87.5% vs. 63.4%, *p* = 0.013). The median time to relapse for those non-transplanted patients was 100 days. The study included patients from age 2–61. It would be useful to compare the outcome in young patients with those in adult patients. It is unclear whether age makes any difference in this type of CAR T-bridging therapy.

Various reports have reiterated that higher CAR T cell doses are associated with better and deeper responses, yet the dose level of the infused CD19-directed CAR T cells in this study was low, which may be responsible for the rapid relapse after CAR T therapy. It was noted that a single-dose level of CAR T cells was used in these patients and majority CR patients proceeded quickly to allo-HSCT. Therefore, the CAR T cells in this report were used as reinduction therapy and allo-HSCT served as a consolidation therapy. This report suggests that the longer survival in these patients who received low-dose CAR T cells was the result of allo-HSCT, and the approach of using low-dose CAR T cell therapy in R/R ALL should only be used as a bridging/reinduction therapy. Due to the limit of the study design, it remains unclear what cell dose would be optimal for pre-allo-HSCT bridging/reinduction therapy.

In addition, it remains controversial as to whether allo-HSCT is routinely needed in those patients who achieve deep MRD negativity. In the long-term follow-up of the 53 R/R ALL patients who received CD19-28z CAR T therapy, 32 patients achieved MRD-negative CR [85]. Among these 32 patients, allogeneic transplantation did not lead to better survival than those who were not transplanted. In another retrospective analysis of 135 R/R ALL patients who received CD19-directed CAR T therapy followed quickly by allo-HSCT, CD19-negative relapse accounted for most relapse after allo-HSCT [88]. This analysis again argues that allo-HSCT may not effectively eliminate the CD19-negative ALL clones that evaded CAR T therapy. These observations suggest that additional graft-versus-leukemia effect may not be achieved by allo-HSCT following effective CAR T cell therapy. Sequential, cocktail, or multi-targeted CAR T cell therapy may be of value.

## CD22-directed CAR T cells

Similar to CD19, CD22 is also widely expressed in B cells and in most cases of B-ALL [89–93]. CD22 is usually retained following CD19 loss which is a common mechanism of relapse after CD19-directed CAR T cell therapy. A phase 1 trial of a new CD22-targeted CAR (CD22-CAR) with a 4-1BB domain was reported [94]. This was a dose escalation trial. The study enrolled 21 children and adults with R/R B-ALL, including 17 who failed CD19-directed immunotherapy. The response was found to be CAR T cell dose-dependent. CR was observed in 73% (11/15) of patients receiving ≥ 1 × 10^6^ CD22-CAR T cells/kg, including all 5 patients with dim or no CD19 expression in the leukemia cells. The median remission duration was only 6 months (range 1–20). Mild CARTox were reported. CD22 antigen loss or diminished density was associated with the relapse.

In a recent update of this study, 43 patients were enrolled with a median age of 17.5 years (4–30 years), 58% of these had CD19-negative disease, and 91% failed prior CD19-directed therapy [95]. The updated analysis reported that prior CD22 therapy was associated with decreased CR rates, MRD negativity rates, and shorter duration of remission. Therefore, this study implies that in patients who have failed prior CD19- and CD22-directed therapies, allo-HSCT should be considered earlier rather than waiting till relapse.

## CD19/CD22 dual-targeted CAR T cells

CD19-targeted CAR T cell therapy for R/R ALL has a relapse rate of approximately 50% at 1 year, and the most common mechanism of relapse is due to CD19 antigen loss. To reduce the possibility of relapse due to target antigen loss and /or mutation, dual antigen targeted CAR T cells are being developed and clinical trials have started. Dual-targeted CAR T cells can be generated either with bi-cistronic CARs that express CD19 and CD22 ScFv simultaneously in every cell or with mono-CARs that express CD19 and CD22 ScFv separately. In the latter approach, not all cells may contain both targets, and even in those cells with both CARs, the ratio of CD19 and CD22 CARs may not be 1:1. In the mono-CAR approach, the CAR T cell product is usually a mixture of three CAR T cell populations, namely, CD19-, CD22-, or CD19/22-targeted CAR T cells.

### Bi-cistronic CAR19 × 22 dual-target T cells

One group constructed a bi-cistronic retroviral vector encoding dual CARs against CD19 and CD22 [96]. These CARs contain an OX40 co-stimulatory domain for the CD19 CAR and a 4-1BB domain for the CD22 CAR. To combat the low intensity of expression of CD22 in R/R ALL cells, a penta-valent CD22 CAR was designed and expressed in the bi-specific CAR T cells. The product, AUTO3, was tested in a phase I/II study evaluating the safety and efficacy of the CAR T cells designed to simultaneously target CD19 and CD22 [96]. In the latest update at 2018 ASH annual meeting, 13 patients of 4–16 years of age with R/R ALL were enrolled and 10 received AUTO3 cells. It was successful to generate a product in all 13 patients. The median transduction efficiency was 16% (range 9–34%). These patients were heavily pretreated already (median 3 prior lines of therapy), with 7 patients having failed prior allo-HSCT. One patient failed prior anti-CD19 CAR T cells, and one failed blinatumomab. Ten patients were evaluable after a minimum of 4 weeks’ follow-up. There was no dose-limiting toxicity (DLT) observed to date. Grade (gr) 1/2 CRS was seen in seven of the patients. Four gr 1 and one gr 3 neurotoxicity were reported. The RR was 90% with 9/10 patients in MRD-negative CR, and 100% MRD negativity in the 6 patients who received cell dose ≥ 3 × 10^6^ CAR T cells/kg. The CAR T cell dose appears to be important, since 3 patients who had doses < 3 × 10^6^/kg had only transient responses whereas the 4/6 patients treated at the higher dose of 3 × 10^6^ CAR T cells/kg achieved durable remission with an MRD-negative CR with the longest follow-up of 4 months. This report of early data provided valuable clinical experience for dual-CAR containing T cell therapy of R/R ALL. With this bi-specific CAR T therapy, there has been no relapse with loss of CD19 or CD22 antigen to date. In addition, the penta-valent design of CD22 ScFv is of interest since this will provide further insights as to whether the new design adds additional benefit to clinical outcome.

Hossain et al. also developed a bi-cistronic CAR construct encoding CARs targeting both CD19 and CD22 simultaneously with intracellular signaling domains incorporating 4-1BB and CD3ζ (CD19/CD22.BB.z) [97]. A single institution phase I dose escalation study was initiated for adult patients with R/R B-ALL or NHL after standard therapies. The primary endpoint was feasibility of manufacturing the bi-specific CAR T cells and safety at three dose levels (1 × 10^6^ CAR T cells/kg, 3 × 10^6^ CAR T cells/kg, 1 × 10^7^ CAR T cells/kg). Efficacy was a secondary endpoint. Six patients have been treated, all at dose level 1. All six patients developed CRS, 4 with gr 1 and 2 with gr 2, whereas CRES was observed in 3 patients. All treated patients showed persistent B cell aplasia. One of the two patients who achieved CR was an ALL patient with disease in the bone marrow/blood/CNS. This patient was MRD negative at days 28 and 60. This adult phase I trial of CD19/22 dual-targeted CAR T cells showed feasibility of manufacturing the bispecific CAR T cells and demonstrated preliminary efficacy. Further dose escalation is ongoing.

Schults et al. constructed a bivalent CAR targeting both CD19 and CD22 with a 4-1BB costimulatory endodomain [98]. This bivalent CAR T cell product was tested in pediatric patients in a phase I trial. The primary objectives are feasibility of dual-targeted CAR T cell production and safety at 3 dose levels (1 × 10^6^, 3 × 10^6^, and 1 × 10^7^ CAR T cells/kg). This initial report enrolled four pediatric patients (age 2–17) with R/R precursor-B ALL, and all four patients were treated at cell dose level 1. It appeared that all the four subjects had relatively low leukemia burden. The CARTox included 3 gr I/II CRS and 2 patients with gr I CRES. All 4 patients achieved CR at day 28 after the bispecific CAR T therapy, with 3 MRD negative. In conclusion, the first dose level of the CD19/22-bispecific CAR T cells was well tolerated and effective.

### Cocktail CAR19 and CAR22 T cells

In a recent report by Yang et al., a bi-specific CAR T product was manufactured by transducing autologous T cells sequentially with CD19 CAR and CD22 CAR constructs [99]. The CD22 CAR also includes a ScFv of human PD-L1 in an attempt to reduce CAR T cell exhaustion. A phase I clinical trial was done in patients with R/R ALL. Manufacturing feasibility and toxicity were the primary end points. Nineteen patients were treated with the cocktail CAR T cells in the 2018 ASH Annual Meeting (15 at the time of abstract submission). All 19 patients had CAR T cells successfully manufactured. The enrolled patients received a median number of 1 (0.9–5) × 10^5^ CD19 CAR+ T cells/kg and a median number of 0.36 (0.4–12) × 10^5^ CD22 CAR+ T cells/kg. On day 30 after CAR T infusion, 18/19 (94.7%) cases achieved CR/CRi, with 94.4% of them MRD negative. There were low rates of CRS and CRES. Fourteen of the 19 patients proceeded quickly to allo-HSCT (median time 61 days). The median OS was 236 days (45–515), and PFS 234 days (14–337). There was no relapse among patients who were bridged to allo-HSCT, whereas 3 of 4 non-transplanted relapsed. All relapsed patients had CD19+/CD22+. This study raised several intriguing issues: (1) the infused number of CAR T cells were lower than those from most reports in the literature, which may have accounted for low CARTox, and high relapse rate in those patients without quick bridging to allo-HSCT. This was evidenced by the fact that the relapsed cells retained expression of CD19 and CD22 antigens. (2) The low CAR T cell doses were used in this fashion more like reinduction therapy, and the intension of the cocktail CAR T therapy was not curative and was to bridge patients quickly to allo-HSCT as a reinduction therapy. It remains unclear what the actual composition of the mixed CAR T cells was infused and whether the cocktail CAR T cells are better than the approach with adequate dose of CAR19 T cells, particularly when such low doses of CAR T cells of each type were used. It is neither clear that if the cocktail CAR T cell doses are escalated to higher and more effective levels to aim for durable remissions, whether immediate allo-HSCT is still needed.

Another group established CD19/22 dual targeting CAR T cells by transducing T cells with two separate lentiviral vectors that direct the expression of two separate CARs targeting CD19 and CD22. This approach led to a cocktail of three distinct populations of CAR T cells (anti-CD19, anti-CD22, and anti-CD19x 22). A phase I clinical trial of this cocktail was done in patients with R/R ALL [100]. Among the seven subjects (ages 1–26 years) enrolled, 4 received dose level 1 (1 × 10^6^ CAR T cells/kg) and 3 received dose level 2 (3 × 10^6^ CAR T cells/kg). Among the CD8+ CAR T cells, there were 21.6% with CD19 CAR, 37.8% with CD22 CAR, and 40.6% with dual CD22xCD19 CAR. Interestingly, the predominant CAR T cell population contained CD19 CAR, with median peak values for CD19 CAR, CD22 CAR, and CD19 × CD22 CAR T cell populations of 9.1%, 1.2%, and 2.4%, respectively. Five of the 7 subjects achieved CR, 4 of which were MRD negative. The rest of the two subjects had no evidence of CAR T cell engraftment. No DLT was observed. Only grade 1 CRS was observed in the five subjects. This study reported the distribution of a cocktail of 3 distinct populations of CAR T cells. Selective in vivo expansion of the CD19 CAR T cell population was discovered. It remains unclear what the mechanism is for the predominant expansion of CD19 CAR T cells over the CD22 and CD19/22 dual-targeted CAR T cell populations.

### Sequential administration of CAR19 and CAR22 T cells

In addition to bi-specific CAR T cells, sequential administration of CAR19 and CAR22 T cells was also reported in R/R ALL in a single-center and single-arm clinical trial, which was registered with Chinese Clinical Trial Registry (ChiCTR, number ChiCTR-OPN-16008526) [101]. The CAR T cells contained third-generation CARs. At the data cut-off on April 30, 2018, the median follow-up was 7.6 months (range 1.3 to 22.2 months) for B-ALL. CAR22 and CAR19 T cells were given sequentially in divided doses (range 1 to 4 × 10^6^/kg for CAR22, and 1 to 5 × 10^6^/kg for CAR19). Eighty one patients received CAR22 T cells followed by CAR19 T cells, 8 received CAR19 T cells followed by CAR22 T cells. Fifty of the enrolled R/R ALL patients were evaluable. Among them, 48 (96.0%) achieved CR/CRi at the day 30 assessment, with 94% MRD-negative. The PFS for all B-ALL patients was 12.0 months, and the median OS has not been reached. A total of 23 patients relapsed without antigen loss for CD19 and CD22. Forty-seven patients (92.2%) experienced CRS with 11 (21.6%) at grade 3 or higher, one of which was grade 5. CRES was seen in 7 patients (13.7%). This study indicated that sequential administration of third-generation CD22 and CD19 CAR T cells is feasible and safe. In the patients with R/R ALL, high MRD negativity was achieved. The kinetics of the two CAR T cells in vivo is still unclear, and the follow-up remains short. The durability of the remission therefore awaits longer observation.

## Donor-derived allogeneic CD19-targeted CAR T cells

Patients with R/R ALL relapsed after allo-HSCT have even worse prognosis, and donor leukocyte infusion can only rescue a small proportion of patients. Therefore, the efficacy and safety of donor-derived CD19-targeted CAR T cells for relapsed B-ALL after allo-HSCT were studied.

In a recent report at the 2018 ASH Annual Meeting, 6 patients with R/R B-ALL after allo-HSCT (5 sibling matched, 1 haplo-identical) were enrolled to receive donor-derived CAR T cells containing CD19 ScFv (HI-19 clone) and a 4-1BB-CD3ζ signaling domain. There were 2 patients with full relapse and 4 patients with MRD+ disease [102]. The cell doses were 1.25–3.5 × 10^6^/kg which were infused over 2 or 3 days. All 6 patients experienced mild CRS of grade 1/2. There was no acute or chronic graft-versus-host disease (GVHD). With a median follow-up of 243.5 days, all the 6 patients were alive with complete donor chimerism and remained in MRD-negative remission.

Following the similar approach, two cases of CML patients with R/R lymphoblastic crisis were treated with CD19-targeted donor-derived CAR T cells [103]. Both patients relapsed after allo-HSCT. One of the two patients developed grade 4 CRS and required steroids and tocilizumab. Both patients achieved molecular remission after the donor-derived CAR T therapy. Therefore, donor-derived CD19-directed CAR T cell treatment is a viable option for patients with R/R B-ALL who relapsed after allo-HSCT.

CD123 is a surface marker associated with AML leukemia progenitors [104]. Recent studies have shown that CD123 is highly expressed in the relapsed ALL patients [105]. Dual-targeted CD19 × CD123 CAR T cells have been shown to be effective in mouse models to eliminate CD19 antigen loss after CD19-targeted immunotherapies [106]. In a recent report, donor-derived fourth-generation CAR T cells targeting CD19 and CD123 respectively were administered to three R/R ALL patients who relapsed after allo-HSCT (NCT03125577) [107]. These three patients had relapsed CD19+CD123+ B-ALL after HLA-matched sibling HSCT. Among them, two patients had p190 BCR-ABL-positive ALL with T315I mutation. These two patients were refractory to ponatinib. All three patients had failed chemotherapy and DLI. The donor-derived T cells were transduced with an apoptosis-inducible, safety-engineered lentiviral CD19 or CD123 scFv CAR fused with the following intracellular signaling domain: CD28/CD27/CD3ζ-iCasp9 (4SCAR19 and 4SCAR123). Therefore the CAR T cells carried a fourth-generation CAR with inducible caspases. The infused cell doses ranged 0.26–1.38 × 10^6^ cells/kg. All three patients achieved complete remission after CAR T cell infusions. The two BCR-ABL+ patients remained in complete molecular remission and disease-free for 7 months and 11 months, respectively. The third patient was in MRD-positive but morphological remission at 7 months. It was noted that the MRD+ patient received the lowest dose of CART infusion, 0.26 × 10^6^/kg. Regarding the safety profile of the 4SCAR design, there was no CRES; neither was there greater than grade 2 CRS and severe myelosuppression. Thus, the safety profile of the fourth-generation donor-derived CAR T cells targeting CD19 and CD123 were satisfactory, and the administration of double 4SCAR19/4SCAR123 T cells successfully rescued the high-risk relapse of R/R ALL.

Donor-derived allogeneic CAR T therapy has been explored in multiple trials and for multiple disease types [108]. GVHD rate remains very low in this scenario, possibly due to the low-dose CAR T cells infused. Currently, most trials of donor-derived CAR T cell therapy have been for R/R ALL or other R/R malignancies. It would be interesting to study low-dose donor-derived CAR T cells for consolidation after allo-HSCT in high-risk MRD+ diseases.

## CD19-targeted universal CAR T cells

Two CD19-directed CAR T cell products have been approved for clinical use. These products are from patient-derived autologous T cells. These CAR T cells therefore are made case-by-case with a costly and lengthy production process. Currently, production failure has been reported [49, 85]. In addition, once there is antigen loss or antigen mutation, new CAR T cells have to be made again. Therefore, universal off-the-shelf ready-to-use therapeutic CAR T cells are needed. Genome-editing technologies such as TALEN (Transcription activator-like effector nuclease) and CRISPR-Cas9 are commonly used these days to modify genes and re-engineer cells [58, 59, 109–114]. In addition to the potential for wide application of these cells to multiple recipients, these universal CAR T cells have the potential for targeting multiple antigens without re-editing and production of T cells.

Using the TALEN technology, HLA-A locus, TRAC, was knocked out to prevent TCR-mediated recognition of HLA antigens in an attempt to minimize or eliminate GVHD. To enhance lymphodepletion of recipient T cells, the CD52 gene was also knocked out in the off-the-shelf donor T cells [57]. Alemtuzumab can then be used to deplete recipient T cells without affecting CD52-negative donor CAR T cells. As an example of successful translation of new gene-editing technology from bench to bedside, such TALEN-edited universal CAR T cells targeting CD19, UCART19, have been applied to successfully treat two infants with R/R B-ALL [57]. The two infants of 11-month-old and 4-week old had high-risk R/R pre-B-ALL, relapsed after allo-HSCT, and both had already failed blinatumomab and a variety of rescue chemotherapies. Both patients responded to the universal CAR T therapy with complete remission and successfully received a second allo-HSCT.

**Fig. 1:**
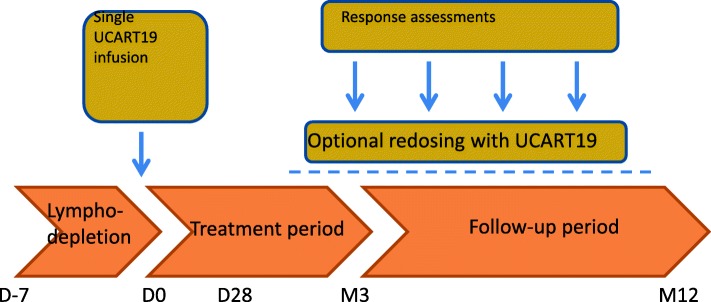
The UCART19 study schema for the universal CAR T cell trials for relapsed/refractory acute lymphoid leukemia (R/R ALL). Two international multi-center studies of the universal CAR T cells, UCART19, are ongoing. PALL is the study for pediatric patients (NCT 02808442), and CALM study is for adult patients with R/R ALL (NCT 02746952). The study schema was modified from the data presented at the 2018 ASH Annual Meeting by Benjamin et al. [115]. D day, M month

Two international, multi-center phase I clinical trials of the off-the-shelf UCART19 cells in adults (from 16 to < 70 years, NCT02746952, CALM study) and pediatric (from 6 months to < 18 years, NCT02808442, PALL study) patients with R/R CD19+ALL have been initiated. To be eligible, all patients must have exhausted available treatment options. The R/R ALL patients must have a morphological disease (> 5% blasts) or MRD load ≥ 1 × 10^−3^. For both studies, the primary end points are safety and tolerability. In addition, in CALM study, additional primary end points include MTD and lymphodepletion regimen. The secondary end point is remission rate at day 28. Patients with extra-medullary involvement are excluded. The lymphodepletion regimens are summarized in Table 1. The study algorithm is shown in the schema (Fig. 1). In the CALM study, 3 CAR T cell dose levels were planned (Table 2). In the PALL trial, there is only one weight-based dose level which may range from 1.1 to 2.3 × 10^6^ cells/kg.

**Table 1:** Lymphodepletion (LD) regimen in the universal CAR T trials PALL and CALM

LD regimen (FC or FCA)	Doses in PALL	Doses in CALM
Fludarabine (F)	150 mg/m^2^	90 mg/m^2^
Cyclophosphamide (C)	120 mg/kg	1500 mg/m^2^
Alemtuzumab (A)	1 mg/kg (capped at 40 mg)	1 mg/kg or 40 mg

The regimen and chemotherapy doses were based on data presented at 2018 ASH Annual meeting by Benjamin et al. [115]

*PALL* UCART19 trial in pediatric patients, NCT 02808442; *CALM* UCART19 trial in adult patients, NCT 02746952

**Table 2:** The UCART19 cell doses in the universal CAR T trials PALL and CALM

PALL dose	CALM dose levels	
weight-based dose (1.1 to 2.3 × 10^6^ cells/kg)	DL1:6 × 10^6^	≈ 1 × 10^5^ cells/kg
	DL2:6 or 8 × 10^7^	≈ 1 × 10^6^ cells/kg
	DL3:1.8 or 2.4 × 10^8^	≈ 3 × 10^6^ cells/kg

The cell doses were based on data presented at 2018 ASH Annual meeting by Benjamin et al. [115]

*DL* dose level; *PALL* UCART19 trial in pediatric patients, NCT 02808442; *CALM* UCART19 trial in adult patients, NCT 02746952

In the recent update at the 2018 ASH Annual Meeting, the data from the two trials were pooled and analyzed together [115]. In the oral presentation at the meeting, 21 patients were enrolled and received a single dose of UCART 19 cell infusion. CR/CRi was 66.7% (14/21 patients). Four of the patients did not receive alemtuzumab (FC regimen) and failed to have UCART19 expansion. These four patients did not respond to the treatment. Among the rest of the 17 patients who received the alemtuzumab-containing FCA lymphodepletion regimen, 14 patients responded, CR/CRi was 82%, and 71% (10/14) achieved MRD. Three patients who were refractory to or relapsed after initial UCART19 infusion received a second dose of UCART19 cell infusion. Two of these 3 re-dosed patients achieved MRD negativity. CRS was observed in 17 patients. Mild to moderate CRS was reported. One patient died in context of severe grade 4 CRS and neutropenic sepsis. Six patients had mild gr 1 and 2 CRES. Two patients (1 infant and 1 adult) developed grade I acute skin GVHD which was easily controlled. Some patients proceeded quickly to allo-HSCT; therefore, UCART19 persistence could not be reliably assessed beyond days 42–56 in those patients after myeloablative conditioning therapy.

In conclusion, in this pooled analysis of the UCART19 clinical trials, only 2 of 21 patients developed grade 1 cutaneous GVHD. This unusually mild and low incidence of GVHD is truly encouraging. CRS and CRES were mostly mild. With the FCA lymphodepletion regimen, high rates of CR/CRi and MRD negativity were achieved after UCART19 infusion in these heavily-pretreated patients who would otherwise have no treatment options. These international multi-center off-the-shelf universal UCART19 trials are currently ongoing. More clinical trials of CRISPR/Cas9 gene-edited universal CAR T cells are underway (NCT03166878, NCT03229876). Both of these universal CAR T trials were for CD19+ ALL. More updates are expected to come in the near future.

## Conclusion

Two autologous CAR19 T cell products have been approved. Antigen loss remains a major mechanism of disease relapse. Dual-target CAR T cells against CD19 and CD22 are in multiple clinical trials. The dosage and kinetics of the dual-target and cocktail CAR T cells remain to be determined. It would be interesting to compare the outcome from dual-CAR T therapy with that from single-CAR T therapy. Donor-derived allogeneic CAR T cells are being studied to combat relapsed malignancies after allo-HSCT. UCART19 universal CAR T cells have been shown in early international multi-center clinical trials to be safe with exceptionally low GVHD rate. This off-the-shelf third-party universal CAR T cell product bears the hope and burden of proof for large-scale clinical application of CAR T cell immunotherapy.

## Abbreviations

ALL: Acute lymphoid leukemia
CAR: Chimeric antigen receptor
CRISPR/Cas9: Clustered regularly interspaced short palindromic repeat (CRISPR)–CRISPR-associated 9 (Cas9)
TALEN: Transcription activator-like effector nuclease
